# The Nurse-Physician Relationship During the COVID-19 Pandemic in Shanghai, China: Cross-sectional Study

**DOI:** 10.2196/41729

**Published:** 2023-02-06

**Authors:** Yueping Shi, Pinhua Gu, Qiufeng Wang, Xuelian Zhang

**Affiliations:** 1 Department of Cardiology Songjiang Hospital Affiliated to Shanghai Jiaotong University School of Medicine (Preparatory Stage) Shanghai China

**Keywords:** nurse, physician, collaboration, online survey, nursing, COVID-19, structural equation modeling, equation modeling, nurse-physician, ordinal logistic regression

## Abstract

**Background:**

The nurse-physician relationship is important for the stability of collaboration. The COVID-19 pandemic has put unprecedented pressure on the health care system and has placed greater demands on nurse-physician collaboration. Nurses and physicians often struggle to share mutual responsibility and communicate effectively.

**Objective:**

This study aimed to evaluate the relationship between nurses and physicians during the COVID-19 pandemic and construct a new model combining the attitude and behaviors of the 2 groups to assess various factors’ impacts on job satisfaction and confrontational behavior.

**Methods:**

We conducted this quantitative cross-sectional study to assess the relationship between nurses and physicians based on the attitudes and behaviors toward nurse-physician collaboration. We first investigated the satisfaction of nurses and physicians with their relationship and how they thought the COVID-19 pandemic had affected that relationship. We used an adapted and modified Jefferson Scale of Attitudes Toward Physician-Nurse Collaboration questionnaire that consisted of 17 items under 5 dimensions. Structural equation modeling was used to assess the relationships between domains. Ordinal logistic regression was used to evaluate the relationship between different domains of the questionnaire and the satisfaction of the current nurse-physician relationship.

**Results:**

We included a total of 176 nurses and 124 physicians in this study. Compared to 7.2% (9/124) of physicians, 22.7% (40/176) of nurses were dissatisfied with the current nurse-physician relationship. Most physicians (101/124, 81.5%) and nurses (131/176, 74.5%) agreed that the nurse-physician relationship had become better because of the COVID-19 pandemic and that the public had greater respect for them. However, significantly fewer nurses (59/176, 33.5% vs 79/124, 63.7%; *P*<.001) thought that physicians and nurses were treated with the same respect. Nurses scored significantly higher scores in *caring versus curing* (mean 16.27, SD 2.88 vs mean 17.43, SD 2.50; *P*<.001) and *physician’s authority* (mean 8.72, SD 3.21 vs mean 7.24, SD 3.32; *P*<.001) subscales compared with physicians. The *shared education and collaboration* subscale had a significantly positive relationship with the *nurse’s autonomy* subscale (standardized coefficient=0.98; *P*<.001). Logistic regression showed that 4 subscales (*shared education and collaboration*: *P*<.001; *caring versus curing*: *P*<.001; *nurse’s autonomy*: *P*<.001; and *confrontation*: *P*=.01) were significantly associated with the level of satisfaction of the current nurse-physician relationship.

**Conclusions:**

This study showed that nurses were more dissatisfied with the current nurse-physician relationship than physicians in Shanghai. Policy makers and managers in the medical and educational system should emphasize an interprofessional collaboration between nurses and physicians. Positive attitudes toward shared collaboration and responsibility may help to improve the relationship between the 2 parties.

## Introduction

High-quality treatment needs close collaboration and the integration of professional skills between health care providers [1]. The COVID-19 pandemic has put unprecedented pressure on the health care system and has placed greater demands on nurse-physician collaboration. Collaboration is a joint decision-making process between independent parties involving common ownership of decisions and collective responsibility for outcomes [2]. However, due to different statuses in the respective clinical practices, nurses and physicians often struggle to share mutual responsibility and communicate effectively.

Each entity providing health care services possesses unique skills to achieve the same goal for the outcome of patients. Poor coordination and integration of resources between health care professionals will lead to disagreements between colleagues and, in severe cases, to medical negligence [3]. The traditional hierarchical relationship between nurses and physicians based on physicians’ authority can lead to negative attitudes and a lack of trust [4]. Health care providers should work together to assess the patient’s illness to achieve the best possible outcome. Physicians’ negative attitudes toward nurses probably create tension between the 2 groups, and compared to physicians, nurses showed a more positive attitude [5]. In China, previous studies have identified that job satisfaction was associated with a high turnover rate and psychological distress among health care providers [6-8]. Nurse-physician interaction can directly affect job satisfaction [9]. A study showed that the nurse-physician relationship worsened from 2008 to 2018 in China [10]. In addition, health care providers’ overall psychological health status is worsening, and they were at high risk of anxiety and depression during the COVID-19 outbreak [11-13]. This may affect the already uneasy relationship between the 2 parties.

Studies about physician-nurse collaboration have focused on surveying attitudes rather than behaviors. It remains unclear how the behaviors and attitudes of health care professionals influence the relationship between nurses and physicians. This study aimed to evaluate the relationship between nurses and physicians during the COVID-19 pandemic and construct a new model combining the attitude and behaviors of the 2 groups to assess various factors’ impacts on job satisfaction and confrontational behavior.

## Methods

### Study Design

We conducted this quantitative cross-sectional study to assess the relationship between nurses and physicians based on the attitudes and behaviors toward nurse-physician collaboration. The sample of this study involved both nurses and physicians working in different levels of hospitals in Shanghai, China. We performed a web-based survey by Credamo (Beijing e-Math Modeling Technology Co.) from April to May 2021. Credamo is a professional research and modeling integrated data platform with a sample database of more than 1.5 million participants. Questionnaires were distributed to health care providers through social media and the Credamo platform, and quality control was performed by response validation, prohibition of duplicate responses, and restriction of IP address at the time of response. As for the consent of participation, only those who agreed and volunteered to participate in the surveys had access the questionnaire. We set up test items to ensure the authenticity of the questionnaire. We excluded questionnaires that failed the test item.

### Sample Size

According to the *Statistical Bulletin on China’s Health and Wellness Development in 2019* [14]*,* released by the National Health Commission of the People’s Republic of China, by the end of 2019, there were 3.867 million practicing and assistant physicians and 4.445 million registered nurses in China. Among them, Shanghai had 77,700 practicing and assistant physicians and 97,100 registered nurses, accounting for 2.1% of the nurses and physicians in China. We calculated the sample size with Calculator.net [15] using a 98% CI and a 2% margin of error. The calculated sample size was 280, which was expanded to 300 as we included interns who were not qualified to practice.

### Measuring Demographic Characteristics, Attitudes, and Behaviors

We used an adapted and modified Jefferson Scale of Attitudes Toward Physician-Nurse Collaboration (JSAPNC) questionnaire that added items related to behaviors [16]. The questionnaire consisted of 2 parts. Part 1 was demographic characteristics, including job title, gender, hospital level, education, and specialization. Part 2 was the attitudes and behaviors toward physician-nurse collaboration: (1) *shared education and collaboration* (items 1, 4, 6, 10, 11, 12, and 15); (2) *physician’s authority* (items 5 and 7); (3) *caring versus curing* (items 2, 3, and 14); and (4) *nurse’s autonomy* (items 8 and 9). Additionally, we added several items to indicate the *confrontation* between the 2 groups (items 13, 16, and 17). The participant’s responses were measured using a 7-point Likert scale as follow: (1) strongly disagree, (2) disagree, (3) somewhat disagree, (4) neither disagree nor agree, (5) somewhat agree, (6) agree, and (7) strongly agree. Furthermore, we added some questions about the overall feeling of the nurse-physician relationship and issues related to the COVID-19 pandemic.

### Statistical Analysis

We assessed the statistical significance of differences by chi-square test for categorical variables. We used a *t* test (2-tailed) to compare the means of the 2 groups (nurses and physicians) regarding different domains of JSAPNC, the Shapiro-Wilk test, and Q-Q plots to ensure normal distribution. Pearson correlation was used to measure the relationship between 2 scale variables. Cronbach α coefficient evaluated the reliability of the modified JSAPNC. We used Kaiser-Meyer-Olkin and Bartlett sphericity test to indicate our data’s suitability for structure detection. Confirmatory-factor analysis (CFA) was used to evaluate whether the modified JSAPNC validated the factor structure. Root mean square error approximation (RMSEA), Tucker-Lewis index (TLI), and comparative fit index (CFI) values were reported as model goodness-of-fit criteria. Structural equation modeling (SEM) was used to assess the relationships between domains. Ordinal logistic regression was used to evaluate the relationship between different domains of the questionnaire and the satisfaction of the current nurse-physician relationship. All analyses were performed in R statistical software (version 4.1.0; R Foundation for Statistical Computing). A *P* value less than .05 was deemed as statistical significance.

### Ethics Approval

The study proposal was approved by the ethics board of Songjiang hospital (2019SQ034). The survey was strictly anonymous to protect the privacy of the participants. All the potential participants were provided with information on the objectives and procedures of the research. Consent was informed, and the questionnaire can only be responded to after providing consent. Participation was completely voluntary and there was no compensation.

## Results

### Sociodemographic Characteristics

We included a total of 176 nurses and 124 physicians in this study. Sociodemographic characteristics and general attitudes toward the nurse-physician relationship of participants are summarized in Table 1. In this study, 98.3% (173/176) of nurses and 60.5% (75/124) of physicians were female. The most frequent education level of nurses (98/176, 55.7%) and physicians (81/124, 65.3%) was a bachelor’s degree. Half (88/176, 50%) of the nurses worked in departments other than surgery and internal medicine, and 56.5% (70/124, 56.5%) of the physicians worked in internal medicine. The most frequent job title for nurses (95/176, 54%) and physicians (60/124, 48.4%) was intermediate grade. Most nurses (93/176, 52.8%) and physicians (64/124, 51.6%) worked in tertiary hospitals. Additionally, 47.1% (83/176) of the nurse and 76.7% (95/124) of the physicians were satisfied with the current nurse-physician relationship. Significantly fewer nurses than physicians (59/176, 33.5% agreed vs 79/124, 63.7% agreed; *P*<.001) thought that physicians and nurses were treated with the same respect. The 2 groups both agreed that the nurse-physician relationship had become better because of the COVID-19 pandemic (*P*=.09) and that the public had more respect for medical staff (*P*=.57).

**Table 1:** Sociodemographic characteristics and general attitudes toward the nurse-physician relationship of participants.

Sociodemographic data				Nurse (n=176), n (%)		Physician (n=124), n (%)		*P* value	
**Gender**									<.001
		Female		173 (98.3)		75 (60.5)			
**Education**									<.001
		College and below		76 (43.2)		16 (12.9)			
		Bachelor’s degree		98 (55.7)		81 (65.3)			
		Master’s degree and above		2 (1.2)		27 (21.8)			
**Department**									<.001
		Internal medicine		58 (33)		70 (56.5)			
		Surgery		30 (17)		12 (9.7)			
		Others		88 (50)		42 (33.9)			
**Job title**									<.001
	Intern		10 (5.7)		12 (9.7)				
	Junior grade		64 (36.4)		30 (24.2)				
	Intermediate grade		95 (54)		60 (48.4)				
	Senior grade		7 (4)		22 (17.7)				
**Hospital level**									.045
	Community health care center		47 (26.7)		46 (37.1)				
	Secondary hospital		36 (20.5)		14 (11.3)				
	Tertiary hospital		93 (52.8)		64 (51.6)				
**Are you satisfied with the current nurse-physician relationship?**									<.001
	Satisfied		83 (47.2)		95 (76.7)				
	Not sure		53 (30.1)		20 (16.1)				
	Unsatisfied		40 (22.7)		9 (7.2)				
**How do you think the nurse-physician relationship has become during the COVID-19 pandemic?**									.09
	Better		131 (74.5)		101 (81.5)				
	Not sure		40 (22.7)		20 (16.1)				
	Worse		5 (2.9)		3 (2.4)				
**How do you think the respect toward health care practitioners has become during the COVID-19 pandemic?**									.57
	Better		119 (67.7)		92 (74.2)				
	Not sure		40 (22.7)		27 (21.8)				
	Worse		17 (9.6)		5 (4)				
**Do you think physicians and nurses are treated with the same respect?**									<.001
	Agreed		59 (33.5)		79 (63.7)				
	Not sure		30 (17)		21 (16.9)				
	Disagreed		87 (49.4)		24 (19.3)				

### Individual Items and Reliability

**Figure 1:**
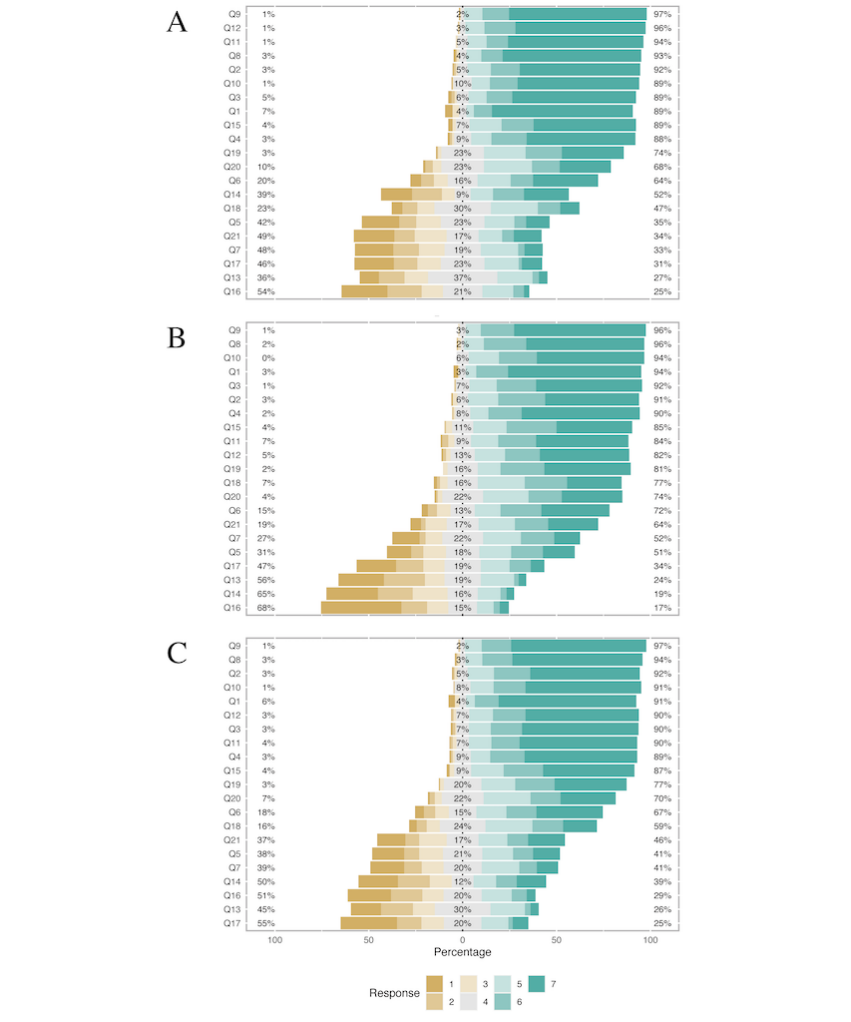
Distribution of the individual items: (A) nurse, (B) physician, and (C) physician and nurse. Q1 to Q17 were the modified Jefferson Scale of Attitudes Toward Physician-Nurse Collaboration (JSAPNC) scales, and Q18 to Q21 were the last 4 questions of Table 1. Q: question.

The complete individual items and factors are summarized in Figure 1 and Table 2. The *physician’s authority* and *confrontation* subscales and *item 14* were reverse-scored. Other items were directly summed based on their Likert weights: the higher the score, the more positive the attitude and behavior toward the nurse-physician relationship. We used corrected item total correlation and Cronbach α coefficients to test the internal reliability of the modified JSAPNC (Table 3). The Cronbach α coefficient indicated that the internal reliability was acceptable. Higher scores meant more positive attitudes and behaviors toward the nurse-physician collaboration. Nurses scored significantly higher scores in the *caring versus curing* (mean 16.27, SD 2.88 vs mean 17.43, SD 2.50; *P*<.001) and *physician’s authority* (mean 8.72, SD 3.21 vs mean 7.24, SD 3.32; *P*<.001) subscales compared with physicians. In addition, the nurse had significantly higher scores in item 11 (*P*<.001), 12 (*P*<.001), 13 (*P*=.001), 14 (*P*<.001), and 17 (*P*<.001).

**Table 2:** Individual items and all subscales of the modified Jefferson Scale of Attitudes Toward Physician-Nurse Collaboration (JSAPNC).

Individual items and subscales	Nurse, mean (SD)	Physician, mean (SD)	*P* value
1. A nurse should be viewed as a collaborator and colleague with a physician rather than their assistant	6.33 (1.47)	6.44 (1.18)	.47
2. Nurses are qualified to assess and respond to psychological aspects of patients’ needs	6.31 (1.15)	6.11 (1.14)	.12
3. Nurses should be involved in making policy decisions affecting their working conditions	6.25 (1.32)	6.24 (1.04)	.96
4. There are many overlapping areas of responsibility between physicians and nurses	6.15 (1.27)	6.31 (1.08)	.25
5. Doctors should be the dominant authority in all health care matters^a^	3.75 (1.94)	4.38 (1.95)	.01
6. Physicians and nurses should contribute to decisions regarding the hospital discharge of patients	5.07 (1.87)	5.40 (1.71)	.13
7. The primary function of the nurse is to carry out the physician’s orders^a^	3.53 (1.88)	4.38 (1.89)	<.001
8. Nurses should clarify a physician’s order when they feel that it might have the potential for detrimental effects on the patient	6.47 (1.12)	6.41 (0.95)	.66
9. Physicians should be educated to establish collaborative relationships with nurses	6.56 (0.89)	6.53 (0.84)	.81
10. Nurses should also have responsibility for monitoring the effects of medical treatment	6.31 (1.11)	6.28 (0.96)	.81
11. You can name most of the nurses/physicians you work with	6.48 (0.95)	5.90 (1.42)	<.001
12. You will ask the nurse/physician for advice at work	6.49 (0.90)	5.88 (1.35)	<.001
13. You have argued with a nurse/physician at work (even if it was just a verbal complaint)?^a^	3.69 (1.50)	3.08 (1.72)	.001
14. When treatment does not work, you will suspect the nurses to strictly follow medical advice/ doubt the physician’s medication or practice?^a^	4.28 (2.25)	2.93 (1.70)	<.001
15. In medical practice, you will point out mistakes made by physicians/nurses	6.06 (1.32)	5.87 (1.20)	.20
16. You have been reprimanded and given a hard time by a senior physician/nurse^a^	3.15 (1.73)	3.56 (1.92)	.05
17. You have thought about leaving the medical profession because of the nurse/physician relationship^a^	3.53 (1.88)	2.63 (1.82)	<.001
Shared education and collaboration	42.91 (5.66)	42.09 (5.51)	.28
Caring versus curing	16.27 (2.88	17.43 (2.50)	<.001
Physician’s authority	8.72 (3.21)	7.24 (3.32)	<.001
Nurse’s autonomy	13.02 (1.73)	12.94 (1.63)	.69
Confrontation	13.62 (3.78)	14.73 (4.26)	.56
Total	94.55 (9.99)	94.43 (10.40)	.43

^a^These were reversed-scored items. When summed to subscales, these scores were reversed.

**Table 3:** Reliability of scales and relationships between scales and subscales.

Scales and subscales	Cronbach α	Corrected item total correlation
Shared education and collaboration	.72	.64
Caring versus curing	.67	.25
Physician’s authority	.63	.36
Nurse’s autonomy	.70	.52
Confrontation	.64	.59
Total	.72	1

### Test of the Model

**Figure 2:**
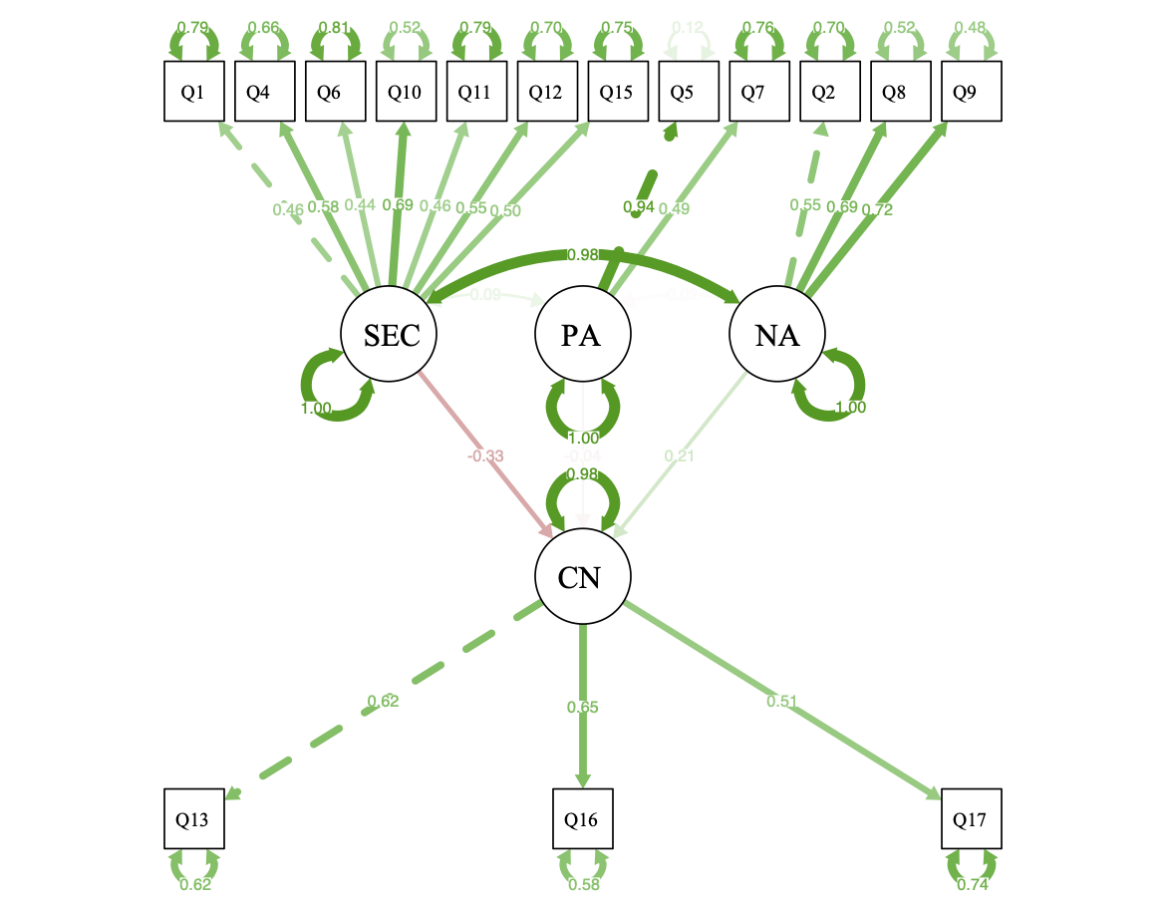
The results of the structural equation modeling. CN: confrontation; NA: nurse’s autonomy; PA: physician’s authority; Q: question; SEC: shared education and collaboration.

We used Kaiser-Meyer-Olkin (0.799) and Bartlett sphericity test (approximate *χ*^2^_16_=1329.081, *P*<.001) to indicate our data’s suitability for structure detection. SEM and CFA were used to analyze the scale’s structural validity and evaluate the model of the modified JSAPNC. When performing the CFA, we found that the *caring versus curing* and *shared education and collaboration* subscales had colinearity. Therefore, we removed the *caring versus curing* subscale and added item 10 to the *nurse’s autonomy* subscale. The adjusted model is presented in Figure 2. The overall model fit indices of the hypothetical model were CFI=0.849, TLI=0.811, standardized root mean squared residual=.061, and RMSEA=.077. RMSEA between .05 and .08 was considered an adequate fit [17]. TLI and CFI values greater than 0.90 were suggested as criteria for an acceptable model fit and greater than 0.95 for a good model fit [17]. Results based on the path coefficients showed that the *shared education and collaboration* subscale had a significantly positive relationship with the *nurse’s autonomy* subscale (standardized coefficient=0.98; *P*<.001)*.* Moreover, the *shared education and collaboration* subscale had a negative impact (standardized coefficient=–0.33, *P*=.9) on the *confrontation* subscale.

### Correlation Between Scales and Demographic Characteristics

In the overall score, female participants scored significantly higher than male participants (mean 84.21, SD 8.63 vs mean 81.10, SD 1.01; *P*=.02). However, in the *physician’s authority* subscale, male participants scored significantly higher than female participants (mean 9.27, SD 3.58 vs mean 7.60, SD 3.21; *P*=.001). In addition, intermediate grade participants scored the lowest on item 17 (mean 1.86, SD 1.35) and highest on the overall score (mean 85.00, SD 10.21). Senior grade participants scored the highest on the *physician’s authority* subscale (mean 9.50, SD 2.14) and the lowest on the overall score (mean 80.88, SD 1.08). There was a U-shaped curve relationship between education and the *confrontation* subscale, with participants with a bachelor’s degree scoring the lowest (mean 7.31, SD 3.04). Participants in tertiary hospitals scored the lowest in the *confrontation* subscale (mean 8.73, SD 2.96). In a subanalysis that divided attitudes and behaviors, the results were similar. We conducted a sensitivity analysis that was logistic regression correcting for the covariates of gender, education, job title, and hospital level, which showed that physicians scored significantly lower than nurses on the *physician’s authority* subscale (odds ratio 0.890, 95% CI 0.807-0.977; *P*=.02) and higher on the *caring versus curing* subscale (odds ratio 1.202, 95% CI 1.072-1.357; *P*=.002).

### Scales and Satisfaction

Ordinal logistic regression adjusted by sex, education, department, job title, and hospital showed that 4 subscales and the total score had a significant correlation with the level of satisfaction of the current nurse-physician relationship (Table 4).

**Table 4:** Ordinal logistic regression.

Scales and subscales	Unadjusted OR^a^ (95% CI)	Unadjusted *P* value	Adjusted OR (95% CI)	Adjusted *P* value
Shared education and collaboration	1.710 (1.561-1.889)	<.001	1.754 (1.596-1.945)	<.001
Caring versus curing	1.286 (1.178-1.410)	<.001	1.335 (1.217-1.471)	<.001
Physician’s authority	0.969 (0.903-1.038)	.38	0.963 (0.895-1.035)	.30
Nurse’s autonomy	2.117 (1.819-2.487)	<.001	2.152 (1.843-2.539)	<.001
Confrontation	1.103 (1.020-1.196)	.02	1.123 (1.003-1.223)	.01
Total	1.119 (1.158-1.245)	<.001	1.216 (1.173-1.265)	<.001

^a^OR: odds ratio.

## Discussion

### Principal Findings

The purpose of this study was to construct a new combined attitudinal and behavioral model to assess the nurse-physician relationship in this current historical context and explore ways to improve it from a systemic perspective. This study revealed that nurses had more positive attitudes and behaviors than physicians. Many previous studies [18-20] had shown that nurses had more positive attitudes toward collaboration, and our results showed that nurses scored higher on almost all subscales. Interestingly, however, those who scored lower on the *physician’s authority* subscale (either physicians or nurses) were more satisfied with the current nurse-physician relationship. One possible explanation was that lower scores in this subscale were associated with more acceptance of the status quo and thus more satisfaction. The structural equation model we constructed corroborated this result. *Physician’s authority* showed a negative association with *confrontation*, although not significant. In addition, nurses scored lower than physicians on *caring versus curing*, and the existing literature was divided in this respect [21,22]. We suggest that nurses may be underrepresented in their perception of their role and should recognize that they should be working alongside, rather than just following, physicians when treating patients. A previous study [18] had shown that positive attitudes were associated with the age of the physician and nurse; however, our study did not yield significant associations regarding job title.

Although nurses were more flexible regarding departments and positions than physicians, our results showed that nurses were more likely to know the names of physicians who work together. Additionally, more nurses than physicians indicated that they experienced arguments with their counterparts. Nurses were also more likely to choose to quit because of the discordant nurse-physician relationship. When treatment was not as effective as expected, nurses were more likely to suspect that the physician had a problematic practice. On the other hand, physicians were more likely to perceive that a senior nurse had given them a hard time. Therefore, there were unbalanced perceptions of the relationship between medical and nursing functions on both sides. For item 7, *The main function of nurses is to carry out physicians’ orders*, nurses scored significantly lower than physicians, indicating that nurses held a more disagreeable attitude. With the reform of the medical system and the development of the nursing profession, the functions of nurses are expanding, and the execution of medical orders is becoming a smaller proportion of nurses’ duties. Today, nurses tend to be decision makers in nursing, caregivers of health services, health educators, managers, researchers, and supervisors of medical orders [23-25]. The differences between the 2 sides may be due to the lack of understanding and learning of the modern functions of nurses during the education of physicians before and after entering the clinic [26], which shaped the values and traits of health care practitioners and thus influenced the development of the health care relationship. The pattern of parallel medical and nursing management has resulted in the segregation and deterioration of the nurse-physician relationship. Physicians, as the main revenue-generating group in hospitals, determine their dominance in the health care relationship [27,28].

Hierarchical interactions between physicians and nurses were popular in China as well as worldwide [4,29,30]. Although the social status of Chinese physicians is not as high as that of their counterparts in the West, the relative status between physicians and nurses is similar [31,32]. According to a 2017 national survey, the average turnover rate of nurses in Shanghai was 4.6%, which was the highest in China, and is increasing over the years [32,33]. The turnover intention of physicians was also high, but the actual turnover rate is unknown [34,35]. Physicians and nurses both experience burnout and job dissatisfaction in their daily work [32,36,37]. However, physicians and patients lack respect for nurses in China. For example, some patients will express gratitude toward the physicians rather than the nurses, and some patients will express anger toward the nurses after a dissatisfied treatment rather than toward the physicians [32]. Some physicians look down on nurses and think nurses should merely carry out orders from them [32]. Many reasons contribute to this phenomenon, such as the difficulty of admission, the length of study, and the upper limit of a career. Especially in big cities such as Shanghai, in the same tertiary hospital where a physician and a nurse both have permanent contracts, a physician must undergo at least 8-12 years of study for their doctorate in addition to the standardized residency training to compete for the position, whereas a nurse may have just undergone a 3-year college program. Nevertheless, we should also recognize that the average scores of nurses and physicians were below 4 for all 3 items that constitute *confrontation*, indicating that the confrontational relationship between physicians and nurses was not that intense.

Comparative analysis indicated that many factors were associated with different aspects of the nurse-physician relationships, such as education, prescribed societal roles, cultural norms, and gender [21,38]. Our study showed that education, job title, gender, and hospital level might influence the attitudes and behaviors of nurses and physicians with each other. These attitudes and behaviors further influence the level of satisfaction of the current nurse-physician relationship. Nurses and physicians with an intermediate title scored the lowest in the question of *resignation because of the nurse-physician relationship*, which may be because they were the mainstay in the hospital and take on the most work and communication. We constructed models corrected for covariates to examine the effect of different subscales on satisfaction with the relationship. The results showed that 4 subscales and the total score, except the *physician’s authority* subscale*,* were significantly and positively associated with satisfaction with the health care relationship. Interestingly, confrontational behavior was associated with higher satisfaction. This may be related to the fact that confrontational behavior is associated with more communication. We, therefore, hypothesized that a correct perception of one’s responsibilities would contribute to positive attitudes and behaviors toward the nurse-physician relationship, thereby increasing satisfaction.

In this COVID-19 pandemic, most physicians and nurses felt treated with more respect than before and that the nurse-physician relationship was moving in a positive direction. However, compared to 36.2% of physicians, nearly 70% of nurses did not feel that they were treated with the same respect as their counterparts. Additionally, significantly more physicians than nurses were satisfied with the current nurse-physician relationship. We believed that society, especially physicians, should embrace the idea of mutual decision-making and sharing responsibility. Increasing the collaboration between the 2 parties may help both sides to understand each other better and create a better working environment.

### Limitation

This study was conducted in only one city, Shanghai. The questionnaire was distributed on the web so the level of hospitals may not have been randomly distributed. The distribution of the hospitals where the participants were located was different from the distribution of hospitals in Shanghai in reality. In Shanghai, there are more community health care centers than other levels of hospitals, but in this study, only 26.7% of the nurses and 37.1% of the physicians were from community health service centers. At the time of this study, the pandemic in Shanghai was relatively stable, and the results in this study do not reflect the nurse-physician relationship during health care system overload. Therefore, the generalization of this study should be limited. Some of the participants in this study were the primary researcher’s colleagues. This could have led to insufficient or untruthful information because of concern over the revelation of personal information even if the web-based survey was completely anonymous. In addition, when conducting the SEM analysis, we removed the subscale of *caring versus curing*, which may have impacted the reliability and validity of the adjusted scale.

### Conclusion

This study showed that nurses were more dissatisfied with the current nurse-physician relationship than physicians in Shanghai. Policy makers and managers in the medical and educational system should emphasize an interprofessional collaboration between nurses and physicians. Positive attitudes toward shared collaboration and responsibility may help to improve the relationship between the 2 parties.

## Appendix

Multimedia Appendix 1 Processed data set.

## Abbreviations

CFI: comparative fit index
CFA: confirmatory-factor analysis
JSAPNC: Jefferson Scale of Attitudes Toward Physician-Nurse Collaboration
RMSEA: root mean square error approximation
SEM: structural equation modeling
TLI: Tucker-Lewis index

## Data Availability

The processed data set is attached as Multimedia Appendix 1. Raw data are available from the corresponding author upon reasonable request.
